# Running out of farmland? Investment discourses, unstable land values and the sluggishness of asset making

**DOI:** 10.1007/s10460-015-9679-7

**Published:** 2016-02-01

**Authors:** Oane Visser

**Affiliations:** International Institute of Social Studies (ISS), Erasmus University Rotterdam, Kortenaerkade 12, 2518 AX The Hague, The Netherlands

**Keywords:** Farmland, Investment, Value, Financialization, Commoditization, Natural resources, Finance, Asset, Land grab, Russia, Ukraine

## Abstract

This article critically analyzes the assumption that land is becoming increasingly scarce and that, therefore, farmland values are bound to rise across the globe. It investigates the process of land value creation, as well as its flipside: value erosion and stagnation, looking at the various mechanisms involved in each. As such, it is a study of how the financialization of agriculture affects the process of land commoditization. I show that, for farmland to be turned into an asset, a whole range of conditions have to be fulfilled, presenting a typology of asset making in the context of farmland. Asset making, like commoditization, is a process of assemblage, and it is less straightforward and less stable than generally assumed. Further, I argue that ‘asset making’ is not a one-way process. The article is based on an analysis of global data on land values and the case of farmland investment in post-Soviet farmland (Russia and Ukraine).

## Introduction

Since the food price hikes of 2007/2008 and the global financial crisis, farmland and the agricultural sector have attracted rapidly growing attention from a financial sector looking for new and supposedly less risky ways to make money. The financial industry’s new appetite for farmland has fueled the so-called global land rush or land grab. While academic research focused initially at states acquiring farmland abroad, and the most visible private actors, attention for the role of finance in farmland (and food) has grown recently (McMichael 2012; Daniel 2012; Fairbairn 2014; Isakson 2014).

Neo-classical and neo-institutional studies have focused on how to enhance the positive impacts of farmland investment (Deininger et al. 2011). Political-economy research has highlighted its risks, studying the growing role of finance in the context of the transformation of the global ‘food regime’ (McMichael 2012). In these studies, financialization has been interpreted as a deepening of the corporate control over agriculture. In addition to a broad range of macro studies (Clapp 2014; McMichael 2012), some articles have emerged that study particular groups of financial actors in the food system, such as agrofood commodity traders (Salerno this issue), private equity firms (Daniel 2012) and farmland fund managers (Ducastel and Anseeuw this issue).

In-depth studies on financial actors in farmland based on empirical field research are still rare (an exception is Fairbairn 2014). This gap seems to reinforce the tendency that finance’s role in agriculture is “conceived largely on a macro level” (Williams 2014, p. 402), with finance as “the main character [which] is present, and yet largely absent” (Ouma 2015, p. 163). This seems to be true for political economy studies as well as for neo-classical and neo-institutional approaches. Subsequently, there is a danger of *assuming* the expansion and deepening of the ‘universal’ dynamics of ‘capital’—such as ‘accumulation by dispossession’ (Hall 2013)—or the ‘market’—growing efficiency—in the agricultural sector, with research on farmland investment becoming a search for indicators confirming these respective dynamics. Discourses that fit within these assumed dynamics will, subsequently not receive the critical examination they need.

In this article I critically evaluate the dominant narrative for speculating in land. Specifically, I deconstruct the assumption that farmland is becoming an increasingly scarce asset, the value of which is only bound to increase as more investors flock to the sector in search of profits from both increased agricultural production and the appreciation of farmland prices. Interestingly, this narrative is evident across a wide range of ideological perspectives, from neo-classical to neo-Marxist scholars, and among investors as well as activists, although the highlighted causes diverge.^1^ The fact that so many actors invest in land might easily be interpreted as proof that farmland prices rise globally and farmland investment is indeed a profitable business, and the grip of finance on agriculture will become firmer across the globe. Why else would so many actors engage in it?

This article takes a critical look at the above-mentioned idea underlying the land rush. It will investigate the process of land value creation (as well as its flipside value erosion and stagnation), looking at the various mechanisms involved. It examines how investors (attempt to) turn farmland into a financial asset, as an important aspect of financialization in agriculture. This means that, like any other asset in an investment portfolio, it can be abstracted, assessed according to benchmarks, and sometimes even sliced and diced—as a result, becoming increasingly ‘distanced’ from the fixed, localized, and lumpy commodity that land is typically seen as. Financialization is defined here as the process in which various parts of the economy are becoming increasingly influenced by the logic (discourses as well as practices) of the financial sector.

The article makes the case that asset making, interpreted as an advanced, yet distinct, form of commoditization, is not a smooth, clear-cut or universal process that is bound to happen when demand rises and financial investors enter farmland. I show that, for farmland to be turned into an asset, a whole range of conditions has to be fulfilled. Asset making, like commoditization, is a process of assemblage (cf. Li 2014), and it will be shown to be less straightforward and less fixed than generally assumed. The article draws on the groundbreaking article of Li (2014), where she argues that rendering land investible requires active efforts of assemblage. While Li mainly focuses on the intricate process of land becoming an asset, the present article also deals extensively with the flipside of that.^2^ Specifically, I refer to the ‘de-assetisation’, the *un*making or ‘erosion’, of farmland as an asset. For analyzing the latter aspect of unmaking an asset, I draw on very recent—and still sparse—work stressing the ‘unfixity’ of investment in agriculture (Ouma 2015; Williams 2014).

While these publications have stressed such ‘unfixity’, they do not investigate farmland investment empirically through fieldwork.^3^ As Ouma (2015, p. 163) states, “we are yet to develop a more grounded understanding of how farmland/agriculture is being turned into ‘an alternative asset class’”. This article aims to develop a typology of asset making in farmland, building on insights from valuation studies, studies on ‘resource making’, and ‘social science studies of finance’. This typology will then be applied empirically in two ways. First, I will analyze global discourses and representations, based on investor reports and graphs (about land values). Second, like Ducastel and Anseeuw (forthcoming)—based on South Africa -, this article presents an empirical, fieldwork-based case of asset making in farmland. I will provide an in-depth analysis of investor discourses and asset making in the post-Soviet region, particularly Russia and Ukraine—which has been heralded as the re-emerging ‘global breadbasket’ (Visser et al. 2014)—based on multi-sited fieldwork.

This case is particularly relevant, as rapid financialization has taken place in the farmland sector in the post-Soviet region. By 2009, Russia and Ukraine already accounted for most farmland companies listed on the stock exchange in the world (Visser et al. 2012).^4^ Further, this case clearly shows the limits and contradictions of asset making, featuring a drop in farmland values after an early farmland rush period with high expectations of land appreciation and aggressive land acquisitions.

This article has the following structure. The first section lays out the theoretical framework. The next section called ‘a typology of asset making in farmland’, presents five requirements for asset making: scarcity, potential for profit, liquidity, standardization and legitimacy. The third section, presents a critical assessment of the widespread assumption of increasing global land scarcity and a pervasive rise of land values. The next sections demonstrate various criteria of the asset making typology (in particular scarcity and legitimacy) by applying them to fieldwork data from the post-Soviet region. The remainder of the article looks at criteria from the typology that were not met in the real life asset making to explain the subsequent erosion of the ‘assetness’ of post-Soviet farmland. Thus, a section analyses how ‘insufficient scarcity’ of farmland has caused stagnant farmland values and discusses the mismatch between discourses of closing yield gaps, and the failure to do so. Another section analyses how the overoptimistic expectations about closing yield gaps came into existence, showing how a ‘fetishization’ of soil led to an ignorance of other relevant factors. As such, it demonstrates the limits of standardization (another requirement of the typology). The final section concludes.

## Financialization, natural resources, and asset making

This article focuses on the recent process of asset making that is part of the current land rush. However, it should be noted that the commoditization of farmland predates the recent financialization of farmland. Land has been sold, hired, valued, and taxed for many centuries in various parts of the world (particularly in the West). In other cases where farmland acquired by financial investors was previously common property, the financialization of the agricultural sector results in enclosure and previously non-commoditized land being turned into a commodity (White et al. 2012). In many other cases, a certain extent of commoditization (e.g. localized sale of land) predates the current finance-driven land rush. In such instances, the entrance of financial actors does not represent a binary shift (from non-commoditized to commoditized), but rather a change along a continuum to a higher degree of commoditization.

The financialization of agriculture entails, among others things, that land is made into a financial asset, which can be valued, easily inserted and taken out of investment portfolios, and subsequently speculated on by financial investors. In the process, it becomes increasingly abstracted and distanced from the physical land. Asset making can thus been seen as an ‘advanced’ stage of commoditization—or, in the case of a natural resource like land, we might more precisely speak of ‘resource making’ (as a specific category of commoditization). As such, it potentially offers investors additional benefits (such as increased liquidity) over an object that is only commoditized. However, at the same time, it also requires additional work of assembling, as will be explained below.^5^

### Valuation studies

Asset making can be fruitfully analyzed as a process of valuation: assessing, communicating, and—to some extent—producing value. A blossoming field of valuation studies has recently emerged (Çalişkan and Callon 2009; Lamont 2012; Styhre 2013; Vatin 2013), which builds on earlier work in sociology and anthropology (Appadurai 1988). The field is characterized by a focus on valuation as a practice or process, rather than on value as just a feature of an object. An insight emerging from this literature is that the process of valuation requires assemblage work, as it is not a natural process (Çalişkan and Callon 2009; and cf. Li 2014). What we call a ‘natural resource’ is “a provisional assemblage of heterogeneous elements including material substances, technologies, discourses and practices” (Li 2014, p. 589). Of relevance here is also the conceptualization of valuation as consisting of two main processes: *categorization* (often involving standardization), and *legitimation* (Lamont 2012).

Building further on the idea of assemblage, valuation studies argue that valuation does not result from abstract market forces, but from the work of concrete actors, among whom some have a much more powerful role than others in the creation of value (Çalişkan and Callon 2009, p. 387, 389). The present article primarily focuses on this creation process, and in so doing, it also gives ample attention to two major actors within the process: the direct farmland investors (the large farm companies, or ‘agroholdings’ owned by actors outside the agriculture sector) and large farmland brokers/consultants.^6^

### Resource making

While valuation studies provide useful insights in the social construction of value, they give limited attention for how the materiality of an object engenders and limits the construction and subsequent boosting of value. With regard to natural resources like farmland, however, materiality plays a very important role. Although it is important to conceptualize resource making as a human process—in line with Zimmermann’s (1951, p. 15) dictum that “resources are not; they become”—one should not overlook the limits set by the supposedly ‘inanimate’ world to human cognitive and practical remaking (Richardson and Weszkalnys 2014, p. 15). Indeed, as Richardson and Weszkalnys (2014, p. 15) argue, “the ‘becoming’ of resources is now better understood in terms of the uses and possibilities that matter affords to us –what may be referred to as material agency or potentiality, which themselves depend on the historical, social and material environments which inform the constitution of the resource matter.” (cf. Bennett 2010).

Another insight from the field of resource making is that natural resources are “a potent social category into which—and out of which—can slip those parts of the non-human world to which humans attached value” (Bridge 2009, p. 1218). A financial asset, like a resource, is an assemblage which does not automatically have fixity of itself. It can be reassembled and reconstituted, with various components combined into new arrangements (Li 2014). Valuation studies also investigate how objects become commoditized or de-commoditized (Appadurai 1988), but focus on socio-cultural causes. Work on resource making pays more attention to how material changes of the object itself (whether resulting from humans actions or natural causes) and wider economic and technological change affect the value of resources. The process of resource making is not just a move between a dichotomy of non-resource and resource (or vice versa). Instead, the making of a resource and its more ‘advanced’ form of commoditization/‘resourceness’ (becoming a financial asset) are often complex and multi-faceted processes (cf. Ducastel and Anseeuw this issue).

Fundamentally, resource making is a process of abstraction (Richardson and Weszkalnys 2014, p. 13), which includes separation and simplification/reduction, both on the material and the conceptual levels. Physical abstraction may be paralleled by the resource’s homogenization and standardization (e.g. Richardson and Weszkalnys 2014, p. 13). The term ‘(farm)land’ itself is already an abstraction (Li 2014).

An important feature that distinguishes farmland from most other resources (Li 2014) is that it features both a use function (cultivation) and a function as a store of value or source of appreciation. Normally, resources have either a—often one time—productive/use function (e.g. oil) or a function as a store of value (e.g. gold). Farmland, however, can potentially offer both, functioning as a kind of ‘gold with yield’, as some investors have called agricultural land (Fairbairn 2014).^7^

### Sociology of finance

Once a natural object has been constructed as a resource, additional assembling is necessary to transform it into a financial asset. For studying this last step, the sociology of finance—and more broadly, the social science study of finance—offers valuable insights, particularly with regard to valuation in (and by) the financial industry and the construction of liquidity (Carruthers and Stinchcombe 1999). A key requirement in order for a resource (or another object) to become a financial asset is its liquidity. Liquidity can be defined as “the degree to which an asset is a fungible, generalized resource” (ibid, p. 375). Mechanisms that make a good more easily transferrable (i.e. reduce transaction costs), such as secure property rights, those which make a good knowable (categorization), and which increase demand, make an object liquid (ibid, p. 377). Standardization is key to enhancing liquidity, as it makes a good more easily knowable, subsequently more transferrable, and thus tends to increase demand (ibid).

## A typology of asset making in farmland

Agricultural land as a resource has long been excluded from financialization. The local, natural specifics of land and farming impede the abstraction and especially standardization that involve turning a commodity or natural resource into an asset. The production of an asset will be analyzed here as a financialization process, in which an asset is defined as any value recognized as such by financial markets. To get such a ‘stamp’ of recognition, a good (or service) must be framed to fit with the financial market requirements. These requirements are: (1) the *potential for profit* that an object can generate in the future, preferably outperforming the average profits on financial markets; (2) the *scarcity* of an object; (3) the *liquidity* of an object, which should be sufficiently ‘liquid’ to be easily sold when the investors see fit, with a profit based on the asset’s appreciation (cf. Ducastel and Anseeuw, this issue); (4) the *standardization* of an object, which should be comparable to other assets through standardized indicators and benchmarks, in order to determine its value; (5) the *legitimacy* of an object, which should be framed as ‘normal’ and socially acceptable, and at least not considered ‘immoral’, in order for it to be treated as an asset (within the financial sector). The wider context for these requirements is that state regulation is needed to facilitate them. Each of these five points will now be discussed.

### Potential for profit

A key aspect of asset making is constructing a predictable profitability, in other words “clear and defined income streams” (Leyshon and Thrift 2007, p. 100; cf. Ducastel and Anseeuw this issue). In investment projects, historical patterns of profits and appreciation for similar investment objects are typically used to assess the potential for profit (Carruthers and Stinchcombe 1999; Williams 2014, p. 415). As farmland investment is a very new asset class—particularly in emerging economies—this type of historical data is not (readily) available. As a consequence, the profit potential has to be gauged more tentatively, by taking some characteristics of the asset in the making (farmland) and comparing it with the productivity, land prices, and profits of similar kinds of farmland in developed countries where such data is (more) available.

In the process of asset making and value creation, a few key material aspects of the farmland resource play a role in determining its potential profitability. I discuss these aspects below, based on my interpretation and categorization of various investor criteria used in publications such as Savills (2012, p. 14), one of the world’s largest multinational real estate companies.

Firstly, there are some baseline material characteristics of the resource land. Although all aspects of the evaluation of land value are, to some extent, subjective—depending on the type of evaluator/brokers and the benchmarks they use—these baseline material features are most objective. The inherent soil quality is generally seen as the logical first determinant of farmland value. Other aspects of land are how square and flat it is. Location in relation to relevant infrastructure and water availability are other important factors. In evaluating land value, some standardization is necessary (e.g. using a broad soil category or average precipitation) to enable comparisons with farmland elsewhere.

Secondly, the potential for yield increases is assessed. Investors are less interested in high productivity per se as they are in the potential to increase it. This is because land with low productivity (which is therefore low value, or ‘undervalued’) can be turned into productive, high value land. The idea is that great value can be ‘unlocked’ with new technology that outside investors with deep pockets can bring in. This is why agricultural land in the Global South and emerging markets has drawn so much attention. It is these regions—where land is cheap and farms have been (supposedly) inefficient—that feature potentially huge increases in productivity, and thus value.

Comparison becomes even more important for determining yield increases than for baseline characteristics. The key indicator currently in use is the yield gap; it has been made into a main, standardized device for assessing investor potential by the financial industry literature, investor conferences, and reports from international agencies like the World Bank (Deininger et al. 2011), as critically discussed by Li (2014) and McMichael (2012). The difference between the actual and the potential yields is based on a comparison of production localities with similar, standardized baseline material characteristics, such as soil (see also point 4 on standardization below).

Thirdly, a certain scale (or scalability) of the natural resource is deemed necessary for making it an asset. This is because some innovations to increase productivity (and close the yield gap)—such as large machinery—would not be cost effective on small plots, whereas larger plots would enable economies of scale.^8^ Further, buying a resource such as land involves transactions costs, such as costs for evaluating the value and registering land titles. For small plots of land, this would not be a profitable exercise.

### Scarcity

Another aspect relevant to asset making is scarcity. An object can become a commodity without being scarce. For instance, when the prairies were opened up during the westward expansion of the United States, land was abundant; at the same time, it was a commodity, as it was priced and sold. However, scarcity is certainly a factor conducive to asset making. This pertains mainly to assets where appreciation is a major attraction for investors. Farmland, like real estate, is generally included in an investment portfolio because of its potential for appreciation. For a financial asset to appreciate, it should be scarce, or at least widely perceived to be so, in order to have (excessive) demand driving up the price. Although, obviously the Earth’s land is finite, “scarcity rarely takes place due to the natural order of things”. (Mehta 2010, p.3). With imperfect information, decision making by an investor (and other actors) is based on his/her perception of scarcity. Thus, the dominant discourse about scarcity, at that moment, is likely to influence the investor’s choice. Discursive scarcity might be rather decoupled from reality. The supply of a resource (farmland), aside from the (bio)physical amount, is also determined by institutional conditions (such as regulations on land sales). As these depend on policy choices, scarcity is far from a neutral, depoliticized term.^9^ Scarcity, in sum, is a “time-bound and contextual phenomenon” (Mehta 2010, p. 3).

### Standardization

The wider literature on financialization has shown that standardization is an essential property of (liquid) investment (e.g. Williams 2014, p. 415; Çalişkan and Callon 2009) and thus a key element in asset making. As mentioned earlier, agriculture has been excluded from financialization until recently, as the local and natural specifics of land plots make abstraction and standardization difficult. In the past years, however, investment brokerage and consultancy firms (Knight Frank and Citi Private Bank 2011; Savills 2012), and international development agencies and banks—notably, the World Bank (Deininger et al. 2011)—have put together reports, maps, and graphs to facilitate a standardized assessment and subsequent comparison of agricultural areas across the world in terms of, for instance, soil fertility, yield gaps, and land values (cf. Li 2014). These products are tools that provide investors with benchmarks to guide them in the process of selecting investment localities, in what was previously a rather non-transparent and highly localized sector (which is, arguably, still true to a large extent, as will be discussed later on). In this paper’s fourth section, one such comparison tool will be critically assessed.

### Liquidity

Liquidity, or “the degree to which an asset is a fungible, generalized resource” (Carruthers and Stinchcombe 1999, p. 375), depends on the ease with which an asset can be bought and sold. Mechanisms that make assets more easily transferrable (thus reducing transactions costs) and knowable (indirectly leading to lower transaction costs) enhance liquidity, as does anything increasing demand (ibid, p. 377).

First, in the case of farmland’s features, scale is especially important. As mentioned above, there is more demand for larger plots, as they can be worked more efficiently with big machinery, making them easier to sell. Lower relative transactions costs also increase liquidity. Furthermore, large landholdings allow a farm company to be listed on a stock exchange, adding further liquidity.

Second, the broader economic and institutional environment is even more important for liquidity than the features of the land plot itself. Particularly important are the security of property rights and the extent to which the law facilitates its easy sale. International development agencies, such as the World Bank, are lobbying for free land markets. Even with more liberal land markets, farmland remains rather illiquid, if it can only be sold as a physical land plot.

There is a clear link between standardization and liquidity. As Carruthers and Stinchcombe (1999, p. 375) argue, “[v]alue that is difficult to discern, or hard to communicate credibly to a large number of potential buyers, (…) makes an asset illiquid”. Standardization alleviates tends to increase liquidity (ibid).

Third, there are several aspects of contemporary financialization that have the potential to increase the liquidity of farmland. One example is the aggregation of farmland into large landholdings, enabling farm enterprises to issues shares, and even be listed on the stock exchange. This has been taking place in South-America with agribusiness giants such as AdecoAgro, Cosan, and Cresud, which control hundreds of thousands hectares of land and are traded on the New York Stock Exchange or NASDAQ (Fairbairn 2014, p. 15; and personal communication). This has also occurred in post-Soviet agriculture, at an initially even greater pace (Visser and Spoor 2011; Visser et al. 2012). Another mechanism is splitting up farm enterprises into a publicly listed farmland real estate investment trust (REIT) that owns and rents outs the land, and a (non-listed) enterprise operating the farmland (Fairbairn 2014). In those cases a process of abstraction occurs, with ownership increasingly distant and dispersed (cf. Clapp 2014).

### Legitimacy

As previously mentioned, this requirement refers to the extent to which an asset can be framed as ‘normal’, as legitimate (or at least not considered ‘immoral’) when treated as such, which is an important element in creating/establishing value (Lamont 2012). Clearly, this is not an objective criterion. Relevant here is whether investors succeed to frame an investment as more or less legitimate, and to preclude that an opposite framing (as immoral) becomes dominant.

With ‘peculiar goods’ such as objects of nature, the process of turning it into a commodity—let alone making it into a financial asset—can be very controversial (Styhre 2013). An important feature that distinguishes land from most other resources is its strong social (life giving) function (Li 2014). Acquiring farmland in food insecure African countries for export crops or biofuel, is increasingly seen as socially unacceptable in investment circles, due to NGOs’ active advocacy.^10^ Further on, I will show how (and why) farmland investment in the post-Soviet region is considered much more acceptable than, for instance, in Africa.

I will now apply the typology empirically, starting with the criteria of scarcity.

## Scarcity and rising farmland values: analyzing global investor discourse

Global investment reports and conferences construct the idea that land is becoming increasingly scarce and that consequently land values everywhere are bound to rise in value. The Savills report (2012, p. 14), for instance, presents a graph showing the rising number of people in the world per hectare, which is further reinforced by declining stocks of land due to urbanization and climate change. Other factors that are often stressed are dietary changes towards more livestock products in emerging economies, propping up demand for food and land. The rapid economic growth of the emerging economies is used to underline the high pace with which land is becoming scarce. Food price hikes are brought in as further proof of the food shortage and the subsequent pressing need for land. These factors are, in the investor parlance, the ‘market fundamentals’. Furthermore, Mark Twain’s statement: “buy land, they’re not making it anymore,” is frequently quoted in investment prospectuses to lend some literary support to the dry figures, and indicate what should be the simple, logical reaction.

Various critical studies (e.g. McMichael 2012) have shown that changes in such ‘market fundamentals’ as population growth and diet trends are in fact rather gradual, and therefore cannot explain the short-term food price hikes or rising land values (if the latter is indeed true).^11^ However, this evidence is largely absent in the investors’ discourse.

Once the notion of land scarcity is firmly established discursively, it lends support to predictions of a rapid rise in global land values and the subsequent profitability of farmland investment. A Savills report (2012, p. 14) even states that: “Our Global Farmland Index (in US$ per hectare) shows that farmland values across the globe have increased up to 1800 % during the past decade, with the highest growth recorded in the emerging markets”. With no firm data on land values in most countries beyond the West, there is a lot of room for selective interpretation. For instance, the striking figure of 1800 % was only recorded in Romania (ibid), with prices hikes much lower in all other countries. It is unclear how such reports come to a global average of land price growth.

It is, thus, relevant to more closely examine some visual representations of the ‘land rush’ by investment brokers. For instance, the Knight Frank farmland index (see Table 1) suggests impressive annual growth in global land values, based on 2010 price changes. However, a closer look shows that the table presents a highly selective group of cases.

**Table 1:**
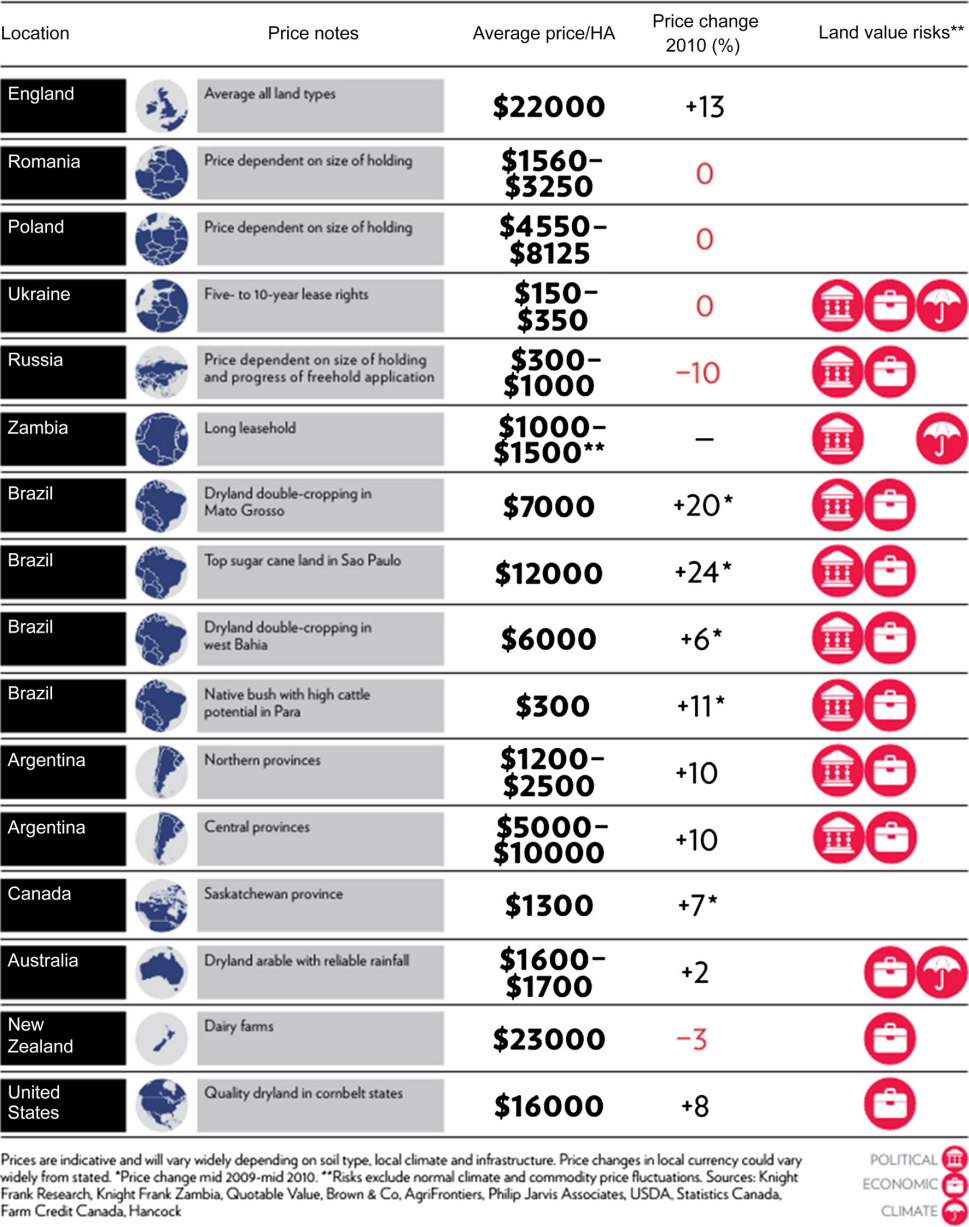
Knight Frank international farmland index

*Source*: Originally published in The Wealth Report produced by Knight Frank Research (Knight Frank and Citi Private Bank 2011)

First, the selected group of countries features several developed countries, only one from the African continent (Zambia), and no Asian countries. Second, most of the countries’ figures do not indicate averages for their farmlands, but only certain, mostly well-endowed, agricultural regions. For instance, the figures for Brazil include “Dryland double cropping in Mato Grosso” or “West Bahia”. For Argentina, only the Northern and central provinces are included. For Canada, only Saskatchewan is included. This is a rather exceptional Canadian region, where investors from outside the province had not been allowed to buy land until recently, which drastically contained the rise of land prices (Sommerville and Magnan 2015). With the abolishment of this regulation, prices have jumped to the level of neighboring provinces. In other cases, land prices are only presented for land with rather good characteristics (e.g. “quality dryland in corn belt states” for the United States, or “dryland arable with reliable rainfall” for Australia). Third, there are serious questions about the reliability of the underlying data, especially for countries in the Global South. Fourth, and finally, in some of the listed countries with high land price hikes, this rise has already flattened or stagnated since the data was collected (in 2009/2010). In Argentina, for instance, due to economic crisis and increased export taxes on agricultural commodities, many outside investors have withdrawn from agriculture. Land prices are still growing only in the prime agricultural areas of central Argentina, and at a much lower pace.^12^ In neighboring Brazil, agricultural production continues to boom—in the soy producing regions—and land prices continue to rise. However, with the recent crisis in the sugar sector, land prices in the sugar producing regions are stagnating, albeit not falling.^13^

My intention here is *not* to argue that the rise of land prices does not occur at all. Rather, I argue that it is not a universal, global, and unilinear trend. The rise of land value is, for instance, clearly happening in parts of Brazil, the United States, and Romania. However, such cases feature a rare conjunction of factors favorable for rises in land prices, which are absent in most other contexts. In Romania, for instance, the initial low land prices combine with a relatively good river infrastructure, ports with easy access to the world market, and a number of EU-related features, such as proximity, and free access, to the EU market, agricultural subsidies, and good legal protection of land rights. Furthermore, Romania’s 2007 accession to the EU coincides neatly with the global land rush. All these factors created an ideal context—or a ‘perfect storm’—for a land price hike. Therefore, the case of Russia would likely be closer to the typical developments of many developing and emerging countries, and therefore more instructive.

To sum up, tables used to depict the farmland rush, such as the one analyzed above, are far from a general reflection of global land price developments. Instead, they are highly selective representations performing a key function in the assemblage of farmland as an attractive financial asset.

While asset making is largely a matter of the imaginary, consisting of narratives, tables, and graphs, I will argue that it cannot persist in the longer term without a base in the material world (real scarcity of land and rising farmland prices). As Williams (2014) and Loftus and March (2015) contend, the materiality of the objects being financialized remains important. In the following sections, based on the example of post-Soviet farmland investment, I show how growing evidence of ‘false scarcity’ and stagnating land prices has begun to undermine the investor imaginary promoting farmland as an asset.

## Scarcity and rising farmland values: post-Soviet investor discourse and institutional conditions

### Investor discourse

I now turn to evidence from the former Soviet region, which—despite being a hotspot of large-scale land deals since 2006 (Visser et al. 2012)—has received little scholarly attention. This section and the following ones will draw on data from Russia (and, to some extent, Ukraine),^14^ whose vast stretches of fertile—including abandoned—land seemed to hold great promise for global and domestic land investors. The analysis is based on web-search, interviews, farm visits, and participation in investor conferences and meetings.^15^

Numerous factors suggest that Russia and Ukraine have, or at least have had, a farmland rush. International and domestic media have discussed it using titles like: “rush for land”, “Russian farms have become hot capitalist property”, “Russian farms could kill off US agriculture” (Visser et al. 2012). Countless web-advertisements offer farmland. Investors’ increased interest in Russia is primarily generated by very low prices and large availability of farmland.^16^ This, the investor discourse goes, is not marginal land as is often the case in, for instance, the supposed ‘land reserves’ of African countries (Visser et al. 2012), but prime farmland, at extremely low cost (e.g. RT 2008). According to Didenko (2009), in some of the fertile regions in Russia’s central black earth area due to the sharp rise in demand ‘free’ land—not yet controlled by large farms and investors- is no longer available. The director of the Russian agroholding Razgulay, stated in 2008 (Paxton and Budrys 2008);

By the end of 2009, all the main agricultural land in Russia will be taken. There will still be some land in the mountains, but there’s not much land left for production with elevators or sugar refineries.

Furthermore, predictions of farmland price trends in Russia were generally very positive (e.g. Indikatory rynka zemli 2008, p. 2).

### The assembling of farmland as an asset: institutional aspects

While this article has so far mostly been focused on discursive aspects of the assemblage of the post-Soviet land rush, the longer-term institutional changes are also important. This is particularly the case in terms of creating *liquidity,* which, in turn, enables the scalability of farmland—as previously discussed, an important aspect regarding the *potential for profit.* The introduction of the new Russian land code in the early 2000 s allowed the free sale of land, reinforcing the process of farmland commoditization that had started with the privatization of farmland in the 1990 s following the demise of the Soviet Union. The financialization of farmland was subsequently enhanced by policies such as the sharp increase of state subsidized and guaranteed cheap credit for private large-scale investment in agriculture from the mid-2000s onwards, the support for the development of domestic commodity exchanges, and the promotion of loopholes in the Russian land code regarding foreign land ownership.^17^ During the agrarian reforms of the 1990s, state farm enterprises were mostly reorganized into corporations, which separated ownership from control/management. This was in contrast to the situation of integrated ownership-control in family farms found in most parts of the world. In Russia, this initially purely formal separation—when the employers and managers became the shareholders—rapidly shifted to a *de facto* separation—when outside investors, including financial actors, began to buy up shares and obtain corporate farms and the related landholdings in one go (Visser et al. 2012).

## Legitimacy: post-Soviet farmland investment not framed as a land grab

Despite having regionally specific elements—such as a stronger focus on the soil fertility (see below)—the investor discourse on post-Soviet farmland shows strong similarities with the previously described global farmland discourse. The diverging factor is that the post-Soviet discourse exists largely uncontested in the public sphere, due to the absence of substantial counter-discourses. In other words, the *legitimacy* of targeting farmland as a financial investment—an important criterion for asset making, as distinguished earlier—has been relatively ‘unproblematic’ as compared with many other world regions.

From media research and interviews with investors, it appears that ‘land grabbing’ is mostly seen as a non-issue. The argument is that land grabbing does not apply to Russia and Ukraine as there is no forced displacement of people, villagers are longing for a continuation of large-scale farming, and the (aging) villagers are willing to sell or rent out their land.^18^ This investment discourse is not significantly contested by societal forces, as farmer movements or NGOs defending the land rights of rural dwellers are weakly developed (Mamonova and Visser 2014). Soviet history has led to distrust with regard to collective action. In Russia, the increased containment of assertive civil movements is another cause. The uncontested celebration of black earth farmland as an asset for profitable and legitimate investment—enabled by the virtual absence of counter-discourses—shows that financial assets like “resources ‘become’ only through the triumph of one imaginary over others” (Bridge 2009, p. 1221). With the ‘triumph’ of the investment discourse over alternatives (e.g. ‘land grab’), asset making is presented as a depoliticized, technical process.

## The erosion of farmland as an asset

### Insufficient scarcity and stagnating land values

Data from news articles and investor reports from 2010 onwards, and from interviews with investors conducted in the period 2011–2014 provide a very different picture than the discourse discussed above, particularly in the case of Russia. In these more recent sources—particularly the in-depth interviews- investors painted a less rosy picture than in the dominant public investment discourse. For instance, an article on Russia’s fertile central black earth states: “there seems to be enough land and profits for all” (Kuzmenko 2012). Moreover, the executive director of Sovecon (a consultancy and research agency), stated: “The bad news is that supply of land in Russia greatly exceeds demand…” (ibid).

In line with this is the following statement of Mikhail Orlov, former director of a Swedish agroholding in Russia: “Given the fact that a huge amount of land is not tilled and is turned into woodland, it’s just not in demand” (ibid). Furthermore, in a 2011 interview, a foreign farm manager at a foreign farm company in Russia indicated:

…it’s by far not as booming as the situation you see in other Eastern European countries, like Romania. (…) prices of land, they might go up. Especially near the cities (…). But if it comes to ordinary farmland, it’s not really a topic.

In the same year, an interviewed foreign farm manager/consultant for Russian agroholdings is even more outspoken: “The rise of land prices in Russia is a myth. There is simply too much of it”. The Russian-American director of the agroholding ‘Russian Farms’ even speaks of falling land prices (RT 2008). Figures on land prices in Russia do also indicate that, after an initial rise in the 2000s, prices have stagnated or even fallen (e.g. Economist 2012), with the exception of Russia’s prime agricultural region, Krasnodar, which is also strategically located near ports for export. Many agroholdings were negatively affected when the global financial crisis hit Russia and Ukraine in 2009 (Kuzmenko 2012), facing more difficulties in getting low interest loans. After some improvement, the recent conflict in Eastern Ukraine and the Western sanctions against Russia have led to economic decline in both countries, negatively affecting agriculture (particularly Ukraine).

In sum, the evidence presented above strongly suggests that demand for agricultural land in most Russian regions is far below the available supply. This might be an important reason for the stagnation of farmland price increases.^19^

Worldwide investors are attracted to farmland largely due to its value (hoping for land appreciation, or at least a robust store of value) with its function as a “means of production only as an afterthought” (Fairbairn 2014, p. 11). The stagnating appreciation of farmland poses a threat to the foundations of the farmland investment model. In the post-Soviet area, the (initial) focus of farmland investors on land appreciation seems to be even stronger than in other regions—such as, for instance, South-America. A study of the largest Scandinavian investors in Russian and Ukrainian farmland shows that, until recently, they cultivated less than 50 % of the farmland they controlled (Kuns et al. 2014). The very low prices of the fertile farmland in the former Soviet Union are an important reason for the one-sided focus on land values among the investors.

### The inability to close the yield gap

In addition to the land values, the yields generated by post-Soviet farmland and the profitability of the farming operations were also far below expectations. Initial yield gap estimations by investors and investment brokers varied, but often indicated that the productivity of Russian farmland could increase by some 50 %, with capital gains of 50–70 % or more (Indikatory rynka zemli 2008). Some investors even spoke about doubling or trebling productivity (Kuns et al. 2014; Lindstedt 2008, p. 181, 226).

However, in reality, none of the large foreign investors in Russian and Ukrainian farmland—some of them 7 or 9 years in operation—have been able to close the yield gap or come anywhere close to it. International investors were awash with money from the stock exchange during the period of widespread optimism about the prospects of post-Soviet farmland (Kuns et al. 2014; Lindstedt 2008; Luyt et al. 2013). Despite large investments in equipment and personnel, the yields of farms operated by foreign investors (ibid) (as well as by Russian and Ukrainian investors from outside agriculture, see Visser et al. 2014) are not performing significantly better than those of domestic (less capitalized) farms without outside investment. Even after a shift away from the initial strategy—predominantly focused on rapid land acquisition with a view on appreciation—to more attention for farming operations, yields and profits from farming operations still fall short of early expectations (Kuns et al. 2014; Luyt 2013).

## Limits to standardization: soil fetishization and asset making

### Initial investor approach: celebration, separation, and abstraction

This section deals with the question of why closing the yield gap proved to be much more difficult than initially expected. The investor discourse regarding Russian and Ukrainian farmland has been characterized by a strong focus on the enormous potential of the black earth soil, separated from other aspects of ‘land’. Investors visiting the black earth regions of the heartland of Russia and Ukraine were impressed by how “amazingly deep” the soil was on this “phenomenally productively land” (Rachkevych 2012) holding “incredible potential” (Pratt 2013). When an agronomist of the Black Earth Farming company showed delegates at a farm forum pictures of the rich black soil, the forum’s audience “groaned with envy” (ibid). The foreign companies operating farms in Russia often have pictures of the black earth soil on their websites. ‘Black Earth Farming’ even features the fertile soil in its name.

The strong focus on the fertility of the soil gives the impression that investors cannot go wrong by investing in the land.

This focus on the soil, in isolation from other features of land and its wider context, is illustrated by Black Earth Farming’s director-founder, Mikhael Orlov. On his ‘road-show’ along the financial centers of Europe to attract investors for his company, he took some of the black earth soil with him. During meetings with potential investors, he showed them the soil, so they could see and feel its prime quality. This aptly illustrates the process of separating one aspect of a natural resource in the process of abstraction that is central to asset making.

One could speak of the black earth soil’s ‘fetishization’, both in the common meaning of the word—“an excessive and irrational commitment to (something)”^20^—as well as, to some extent, in the meaning of commodity fetishization in Marxism—the tendency to objectify value created through social relations, as if it were residing in the produced objects themselves.

While the investor discourse is constructed, it clearly did have *some* foundation in the material reality, if a rather one-sided and weak one. Although black earth soils can also be found in some other parts of the world the cases of Russia and Ukraine are rather unique in that the black earth soils are very deep (and subsequently more fertile), with vast areas of low-priced—and sometimes abandoned—farmland.

Furthermore, the asset making that built on the investor discourse celebrating the black earth soil, was also facilitated by and built on long-standing global discourses on the scarcity of farmland. More specific historical discourses fetishizing the black earth soil date from Tsarist (Moon 2013) and Soviet eras, which resulted in the ignorance of other agro-climatic factors, such as heat and insufficient rainfall, which can reduce agricultural yields (Moon 2013).

### ‘Back to reality’: the biophysical limits of asset making

In recent interviews, various investors in post-Soviet farmland indicated that the importance of soil fertility for agricultural productivity and profits has generally been overstated (cf. Kuns et al. 2014). Referring to the 2006–2007 land rush period, one investor said that his company and other Western investors “were driven by a simplistic view” (cf. Luyt et al. 2013). The prevailing idea, as emerged from interviews, was “land is cheap, let’s go and buy it” and “as much as you can”. “At that time it sounded logical”.

This overly-optimistic view was not corrected by more careful, evidence-based assessments, as farm managers and agronomists had no voice in the boardrooms where the major decisions were made. The top levels of the agroholdings often consist of persons with a background in finance and other ‘real sectors’, instead of agriculture or agribusiness. Among the three Nordic farm companies listed on the stock exchange that operate in the post-Soviet region, none featured members with agricultural experience on their boards during the first years of operation (Kuns et al. 2014).^21^ The executive director of one complained that his advice against the purchase of a large farm—due to its poor agro-climatic features—was ignored by the board, where the view of buying as much land as possible prevailed (Lindstedt 2008). The investor-led farm companies in Russia and Ukraine control huge holdings with up to several hundreds of thousands of hectares and some two thousand employees, which are run in a hierarchical fashion (Nikulin 2005). In such an organizational environment, aberrant views from the farm level could not easily make it to the top.

Over time, investors found out—through a costly learning process—that the general weather conditions and microclimate, and in particular the availability of water, are much more critical for productivity than expected. As an investor stated:

We thought that the black soil is the best thing, (…) that you should go for the black soil regions and this was the big, you know, focus for us when we started. (…) You can have the best soil in the world, but if the temperature goes up over 35 degrees, and soil temperature goes to 60 degrees, then you have no rain for 4–5 weeks in a row, it will kill your crop.

In sum, the initial focus on soil fertility—a clear example of the separation, abstraction, and standardization that are part of resource and asset making in farmland—appears to have been too narrow. In the rare cases where farmland investors took into account precipitation, they used standardized indicators such as average annual rainfall by province, which did not capture the drastic interregional and seasonal variability. This resulted in a shaky foundation for asset making, with a subsequently sharp erosion of farmland as a financial asset once investors, faced with recurrent droughts, recognized that soil fertility was a weak predictor of yields and profits.^22^

## Conclusions

This article set out to investigate one of the little examined, basic premises of the global ‘land rush’—the idea that farmland is running out, land values are rising globally, and farmland is bound to be a profitable business. Mostly, this process of commoditization and resource making is presented as a far-reaching, speedy process, a juggernaut which will continue its course relentlessly unless forcefully resisted.

I first showed that global, macro trends that supposedly lead to land shortages (such as growing population or changing diets) do not automatically translate into rapid commoditization and asset making around the globe. The expectation of a solid, ongoing farmland boom in Russia and Ukraine was based on superficial comparisons and the translation of global trends directly to a particular region. Such yield gap suggesting comparisons were based on highly standardized and very selective assessments of the profitability of farmland investment, in turn largely based on one indicator (soil fertility), which was separated from other aspects of farmland and the wider rural context. The notion of the global land rush following the 2007–2008 food crisis has become so dominant that the different trajectories of farmland investment in various localities tend to be overlooked (cf. Hall 2013).

Second, I showed that commoditization and asset making are not one-way processes, and that land that promises to become a profitable asset may become less valuable and less asset-like overtime. In Russia, the early boom in farmland commoditization and land prices stagnated, or even reversed, almost as quickly as it started. However, while there can be stagnation or a reverse movement, it does not necessarily mean that farmland will fully return to its previous state. Once having been implicated in the process of asset making, it seems likely that some of the transformations/alternations in the function(s) and perceptions of farmland, will linger. To what extent, and in what ways, financialization still looms in the background after a stagnation in asset making is an important question for further research.

I also suggest that it is important to differentiate between commoditization and resource making on the one hand, and asset making on the other. While a financial asset can be seen as an ‘advanced’ stage of commoditization, it is clearly distinct from a ‘simple’ (‘non-assetized’) commodity or natural resource, due its higher degree of standardization and liquidity. Thus, we should indeed be careful when substituting the ‘commodification of everything’ with the ‘financialization of everything’ (Loftus and March 2015, p. 174).^23^ “Rather than being dazzled by” the apparent speed and scale of finance (Pike and Pollard 2010, p. 34, cited in Williams 2014, p. 423), we should carefully study it, not confusing, for instance, the speed or magnitude of farmland commoditization with those of financialization.^24^ Thus, while large stretches of African land are enclosed (commoditized) through large-scale farmland deals (White et al. 2012), the amount of farmland turned into a financial asset seems to be much lower, due to low profitability and liquidity (Visser 2015). In the post-Soviet region I studied, as well as in much of the global North,^25^ the current finance-driven land rush is primarily a process of asset making, and to a lesser extent of enclosure, as much of the land was already privately held, and quite strongly commoditized as it could already be bought and sold.^26^ That being said, I do not want to understate the impacts of large-scale farmland investment and financialization. Also, stagnation in the process of asset making does not necessarily mean a lower local rural impact than in cases where farmland is fully transformed into a financial asset. Financial investors that exit hastily when their farmland investment appears less of an attractive asset than initially expected can leave local people worse off than if they had not entered or, instead, had undergone their full-scale, ongoing investment.

The typology of asset making—and its application to global representations of scarcity and land values in general and the post-Soviet region in particular—shows empirically that asset making is very much a process of assemblage, as some recently anticipated/suggested, and that a range of criteria have to be met before farmland is transformed into a financial asset. It is fruitful to recognize the fact that the financialization of farmland—like financialization in general—is an “unfinished business” (Ertürk et al. 2008), “marked by tensions between its rhetoric and its reality” (Williams 2014, p. 410). Uncritical recycling of popular farmland rush idiom and notions of ‘the world running out of farmland’ blinds us to the multifaceted, variegated, and highly influential processes of farmland investment and financialization as they unfold in reality.

## Footnotes

1 Neo-classical economists stress ‘market fundamentals’ such as population growth and farmland reduction, while critical agrarian and food studies highlight causes such as financial deregulation, speculation, and policies privileging large-scale investors.

2 Li briefly discusses the latter.

3 Williams’ empirical case (the Canadian wheat board) focuses on food commodities not farmland.

4 In the past 2–3 years the number of listed companies in this region has gone down (Kuns et al. 2014) as a result of the stagnating farmland boom discussed further on.

5 This process of asset making and broader financialization is likely to decrease the power of rural actors, with all the multifaceted, contradictory, and potentially very impactful consequences for their livelihoods (cf. Bridge 2009).

6 For an analysis focused on fund managers, see Ducastel and Anseeuw (this issue). Kuns et al. (2014) analyze interaction between direct farmland investors (agroholdings) and indirect investors (e.g. pension funds).

7 However, combining the two is not always easy (Kuns et al. 2014; Visser 2015).

8 There are also dis-economies of scale, related to e.g. monitoring costs. Some innovations (e.g. knowledge and biodiversity based) might be more effective on small farms.

9 Part of scarcity results from exclusion and unequal access (Mehta 2010).

10 This is not to say that such acquisitions no longer occur.

11 Instead, the main influencing factors are the *expectation* of rising food and land shortages, combined with the conversion of farmland from food towards biofuel production, and increased speculation with food commodities.

12 Personal communication with Sebastián Senesi (School of Agronomy-UBA, Argentina) 8 August Milan, Italy.

13 Personal communication with Bastiaan Reydon (UNICAMP, Brazil) 9 July 2015 Utrecht, Netherlands.

14 I focus on the black earth area, where Russia’s and Ukraine’s main agricultural regions are situated. For differences between the two countries and their geographical differentiation in relation to farmland investments, see Kuns et al. (2014).

15 I draw here in particular on fieldwork data concerning actors with a transnational dimension; foreign-owned agroholdings, expat farm managers/consultants, and real estate brokers with foreign clients.Web-search included English and Russian-language—general and investor-oriented media, and company websites. Interviews were conducted with some of the key foreign-owned farming companies, including 4 Scandinavian-owned ones, 2 German, and 2 Dutch ones. Interviewees were the CEOs, and additionally some farm staff and investors. Also several interviews were conducted with Dutch expat farm managers/consultants in foreign-owned and Russian-owned farm companies. Where I speak of ‘interviews’ in this article without literature references, it concerns fieldwork interviews (interviewee identities are not disclosed). The work of Brian Kuns and Michelle Steggerda who conducted some of the (joint) interviews, is gratefully acknowledged. Investor meetings and conferences were attended in Ukraine, Russia, Sweden, Denmark, Germany and the Netherlands. Data collection took place during 2011–2014. For more on the methodology see Kuns et al. (2014).

16 After the demise of the Soviet Union, an estimated 30 million hectares of farmland fell out of production.

17 In Ukraine, repeated promises of lifting the moratorium on land sales stimulated the land rush.

18 Visser et al. 2012 critically reviews this discourse.

19 The drop of global wheat prices since 2010 is another major factor.

20 Definition from http://www.oxforddictionaries.com/definition/english/fetish.

21 Even the *executive* director, often did not have an agricultural background.

22 Economic slow-down caused further ‘de-assitisation’.

23 Also the former should be critically assessed.

24 Even as in practice these phenomena and their effects are not easy to disentangle.

25 A rare exception is the current land rush/enclosure of First Nation land in Canada (Sommerville and Magnan 2015).

26 Even as much of Russian farmland was already privatized and corporatized at the eve of the land rush, enclosure has intensified, as the boundaries of corporate farmland have become less permeable (Visser et al. 2012), e.g. the practice of villagers’ private livestock grazing on marginal corporate farmland is increasingly forbidden by corporate farms.
